# Bone Trans-omics: Integrating Omics to Unveil Mechanistic Molecular Networks Regulating Bone Biology and Disease

**DOI:** 10.1007/s11914-023-00812-8

**Published:** 2023-07-06

**Authors:** Benjamin H. Mullin, Amy B. P. Ribet, Nathan J. Pavlos

**Affiliations:** 1 Bone Biology & Disease Laboratory, School of Biomedical Sciences, The University of Western Australia, 2nd Floor “M” Block QEII Medical Centre, Nedlands, WA 6009 Australia; 2 Department of Endocrinology & Diabetes, Sir Charles Gairdner Hospital, Nedlands, WA 6009 Australia

**Keywords:** Bone, Multi-omics, Trans-omics, Bone biology, Osteoblast, Osteoclast

## Abstract

**Purpose of Review:**

Recent advancements in “omics” technologies and bioinformatics have afforded researchers new tools to study bone biology in an unbiased and holistic way. The purpose of this review is to highlight recent studies integrating multi-omics data gathered from multiple molecular layers (i.e.; trans-omics) to reveal new molecular mechanisms that regulate bone biology and underpin skeletal diseases.

**Recent Findings:**

Bone biologists have traditionally relied on single-omics technologies (genomics, transcriptomics, proteomics, and metabolomics) to profile measureable differences (both qualitative and quantitative) of individual molecular layers for biological discovery and to investigate mechanisms of disease. Recently, literature has grown on the implementation of integrative multi-omics to study bone biology, which combines computational and informatics support to connect multiple layers of data derived from individual “omic” platforms. This emerging discipline termed “trans-omics” has enabled bone biologists to identify and construct detailed molecular networks, unveiling new pathways and unexpected interactions that have advanced our mechanistic understanding of bone biology and disease.

**Summary:**

While the era of trans-omics is poised to revolutionize our capacity to answer more complex and diverse questions pertinent to bone pathobiology, it also brings new challenges that are inherent when trying to connect “Big Data” sets. A concerted effort between bone biologists and interdisciplinary scientists will undoubtedly be needed to extract physiologically and clinically meaningful data from bone trans-omics in order to advance its implementation in the field.

## An Introduction to Bone Omics

Bone is metabolically active tissue that undergoes iterative cycles of bone resorption and formation throughout postnatal life in order to repair and replenish the skeleton [1]. This process, termed “remodeling,” is orchestrated by the coordinated activities of key bone resident cells, i.e., bone-digesting osteoclasts, bone-forming osteoblasts, and mechanosensing osteocytes. Imbalances in bone cell numbers and/or activities feature in a spectrum of skeletal diseases, ranging from metabolic bone-wasting diseases such as osteoporosis, to rare high bone mass disorders like sclerosteosis.

**Fig. 1:**
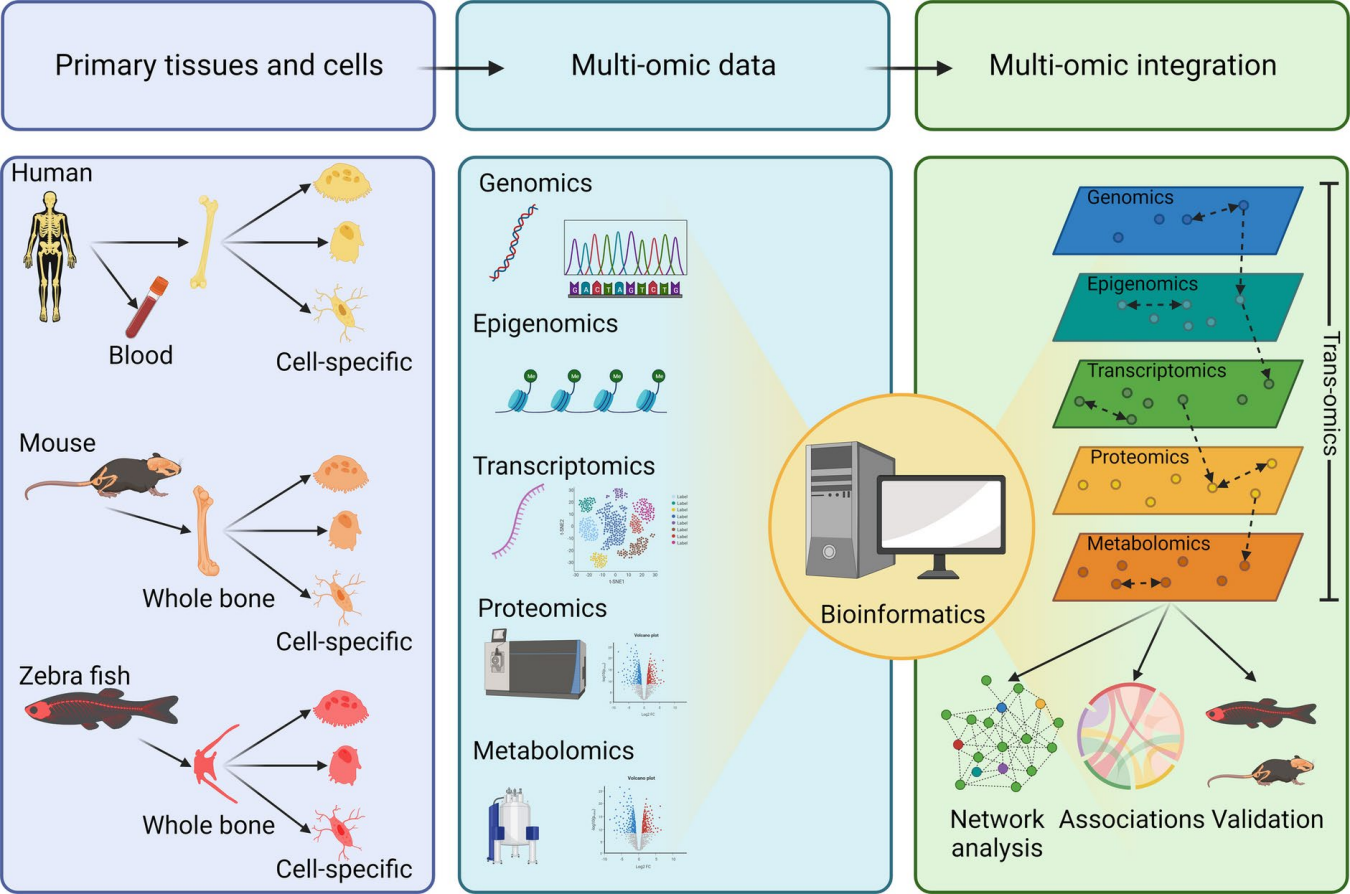
Schema illustrating integration of multi-omic layers (trans-omics) in bone biology. Primary bone tissue, blood, or cells can be isolated either from diseased tissues or model organisms and analyzed in parallel using one of five distinct omic modalities, i.e., genomics, epigenomics, transcriptomics, proteomics, and metabolomics. Multi-omics data sets can then be integrated using bioinformatics and statistical methods. The multi-omic integration depicted flows from DNA to metabolites. Each parallelogram represents an individual molecular omics layer. Circles on the parallelograms represent molecules that belong to their respective layers. Dashed arrows between or within represent molecular interactions that mechanistically connect molecules both horizontally and vertically. These connections can be used to construct molecular networks and tissue/cell atlases to inform mechanistic associations that can be subsequently validated using vertebrate models (e.g., mice or zebrafish). Created with BioRender.com

Being a densely mineralized and complex tissue lined with cellular residents tightly adhered to its surfaces (osteoblasts, osteoclasts) and entrenched deeply within its matrix (osteocytes), bone has long proven a relatively inaccessible tissue for biologists. Much of our current understanding of bone biology has been gathered from unsystematic studies of genetically modified mice exhibiting bone phenotypes and through observations of bone cells cultured in vitro, which are inherently subjective. Recent advancements in bone cell isolation procedures combined with high-resolution “omics” technologies have afforded researchers new methods to interrogate bone biology in an unbiased and systematic way. So far, most bone omics studies have used ‘single’ omics platforms to profile measureable differences (both qualitative and quantitative) of distinct molecular layers, including genes (genomics), epigenetics (epigenomics), RNA transcripts (transcriptomics), proteins (proteomics), and metabolites (metabolomics). While each omics platform has the potential to capture a snapshot in a bone cell’s lifetime or disease state, individually they lack the power and capacity to capture holistic and spatiotemporal changes that occur both at the cell and tissue level [2]. Recently, momentum has grown towards the integration of multi-omics data sets spawning the discipline of “trans-omics” [3]. The definition of trans-omics is continuously evolving but, in this review, refers to findings that span across at least three different molecular omics layers. When combined, these multi-modal omics layers generate deep molecular maps that can be used to discover new pathways, networks, and mechanistic interactions pertinent to bone biology and disease (Fig. 1).

Herein, we summarize recent mechanistic insights gleaned from multi-omics and integrated omics studies in bone. First, we briefly introduce the major omics technologies and provide examples of their individual and combined application in bone research. We then highlight recent studies implementing integrative multi-omics and trans-omics to advance our mechanistic understanding of bone biology and disease. Finally, we outline challenges associated with unifying multi-level omics data sets and offer future perspectives.

### Genomics

The major objective of genomics technologies (e.g., whole genome sequencing (WGS), exome sequencing, genotyping arrays) is to understand genetic causality of complex traits of human diseases. Most complex-disease genomics studies focus on testing for association between variation in the genetic code and a particular trait. Genome-wide association studies (GWAS) have been extremely popular since the first single nucleotide polymorphism (SNP) genotyping arrays were developed in the 2000s. These studies typically involve genotyping a large number of genetic variants distributed across the genome using an array chip, with subsequent imputation of additional variants using a reference genotype panel. Each variant is then tested for association with the trait of interest, with a multiple-testing-corrected significance threshold used to identify significant associations (typically *P* ≤ 5 × 10^−8^). A large number of GWAS have been performed for bone traits, many of which have focused on dual-energy X-ray absorptiometry (DXA) bone mineral density (BMD). Early examples of these studies typically included a few thousand individuals [4], with larger meta-analyses progressively identifying greater numbers of genome-wide significant loci [5–7].

Medina-Gomez et al. [8] performed a GWAS meta-analysis for total-body BMD in a total of 66,628 individuals, identifying 80 genetic loci as associated with the trait. Separating the results into 5 different age strata, the authors concluded that most genetic variants associated with BMD likely influence the trait early in life, with their effects able to be seen throughout an individual’s lifetime. Recently, a GWAS meta-analysis for skull BMD, a non-weight-bearing skeletal site, was completed in around 43,800 individuals, providing an opportunity to focus on the genetics of intramembranous ossification [9]. Fifty-nine genetic loci were identified as associated with the trait, including several harboring genes with a role in craniosynostosis processes. Other GWAS have focused on bone traits derived from quantitative ultrasound measurements at the heel [10–12]. The largest of these studies published to date focused on estimated BMD (eBMD) in a study population of 426,824 individuals from the UK Biobank [13••]. A total of 518 genome-wide significant loci for eBMD were identified in the study, accounting for approximately 20% of the trait variance. The results from these studies emphasize the highly polygenic nature of bone structural traits.

Despite the success of GWAS in identifying large numbers of genetic loci associated with bone traits, in many instances the identity of the effector gene/s driving the associations remains to be determined. As is the case for most complex diseases, the majority of bone trait GWAS signals are led by intergenic or intronic non-coding variants, which likely influence the trait through regulatory effects on nearby genes. Identifying these effector genes is key to maximizing the discoveries made by GWAS. Combining transcriptomic data with the results from GWAS using computational approaches, such as co-localization, summary-data-based Mendelian randomization (SMR), or transcriptome-wide association studies (TWAS), can provide information on genetic regulatory effects that underlie some of the association signals. Expression quantitative trait locus (eQTL) studies can also be used to map the genome to the transcriptome, identifying genetic variants that are associated with the expression of nearby genes [14, 15]. Al-Barghouthi et al. [16] performed a TWAS and co-localization study for eBMD using eQTL data from 49 GTEx tissues, identifying 512 genes as significant using both approaches. Of these 512 genes, less than half (40%) were the closest gene to the eBMD GWAS association signal, demonstrating that the likely causal gene is often not the nearest one.

### Transcriptomics

Considering that regulation of gene expression is largely tissue and cell-type-specific, studies using transcriptomic data from bone tissue/cells are of particularly high value to the bone field. Mullin et al. [17–19] generated a human osteoclast-specific eQTL resource using cells differentiated in vitro from 158 individuals. The authors used this data to identify putative GWAS effector genes for eBMD [17, 18], skull BMD [9], and Paget’s disease [19], implicating several genes linked with osteoclast function such as *IQGAP1* and *CCR5*. Many of the GWAS association peaks appeared to co-localize with more than one eQTL signal in these studies, highlighting the complex molecular architecture underlying some of the bone trait loci. Another study used a systems genetics approach to perform integration of bone transcriptomics and GWAS data in a diversity outbred mouse model to identify genes that regulate bone structure [20]. The authors used the data from this resource to highlight 66 genes potentially underlying human BMD GWAS associations and identified the *Qsox1* gene as driving a QTL for cortical bone morphology in mice.

Differential gene expression analysis techniques using RNA sequencing (RNA-seq) data have also proven highly informative to the bone field in recent years when used to compare or quantitate differences in gene expression between experimental groups and have been reviewed extensively in Chai and Wee et al. [21, 22]. Recently, Rashid et al. [23] compared the gene expression profiles of human osteoclast-like cells differentiated in culture and their peripheral blood mononuclear cell precursors, identifying a large number of genes differentially expressed during osteoclastogenesis. Several biological pathways were identified as enriched for genes upregulated in the osteoclast-like cells, including focal adhesion, lysosome, and metabolic activity, the latter of which likely reflects the high energy requirements of the cell type [24].

### Epigenomics

There is an increasing appreciation that bone traits and phenotypes are determined by interaction between the genome and epigenome. Epigenetic modifications (e.g., DNA methylation, histone modifications, non-coding RNAs), defined as functionally relevant modifications to the genome that do not change the sequence, can have substantial effects on gene expression levels by influencing the binding of regulatory elements to the DNA. One of the best recognized forms of epigenetic modification is DNA methylation, whereby a methyl group is added to the DNA molecule, typically at CpG sites. Morris et al. [25] performed an epigenome-wide association study (EWAS) for BMD using DNA methylation data derived from whole-blood samples from 5515 individuals. They identified one DNA methylation site, cg23196985, as significantly associated with femoral neck BMD in male and female subgroups after correction for multiple testing. However, these associations were not seen in a smaller replication cohort.

Epigenetic data can also be analyzed in conjunction with other -omics data sets in order to gain an understanding of how the different -omes interact with each other to influence disease susceptibility. For example, Yu et al. [26] analyzed DNA methylation in peripheral blood monocytes from 118 women with either high or low hip BMD measurements, identifying 2188 differentially methylated sites between the 2 groups. They then performed an integrative analysis of genotype and DNA methylation data for the cohort, identifying genetic variants associated with nearby methylation sites (known as methylation QTL or mQTL). Using a combination of genetic fine mapping, Mendelian randomization and co-localization approaches, they identified 30 differentially methylated sites potentially mediating genetic effects on BMD.

### Proteomics

Proteomics is the large-scale study of all proteins, termed the “proteome,” that compose an organism, tissue, cell, or organelle under a defined set of conditions. Unlike, genomic and transcriptomic studies, proteomics is considered closer to the phenotype, and is therefore a more reliable approach for biomarker discovery and for investigating disease mechanisms. Over the past decade, proteomics has emerged as a powerful investigative tool for bone biologists. Although far too many studies to credit individually in this short review, there are a number of existing reviews describing the application of proteomics in bone research [27] as well as focused literature documenting proteomic approaches to study osteoblasts [28], osteoclasts [29], and osteocytes [30].

From an osteoclast-centric view, most proteomic studies thus far have employed bulk proteomic methods to profile osteoclast-like cells derived from RANKL-stimulated RAW264.7 macrophages. However, despite their wide appeal and ease of culture, caution is warranted when interpreting proteomic data arising exclusively from osteoclasts derived from immortalized macrophages. This is exemplified in a recent proteomic study by Ng et al. [31], which uncovered major discrepancies in “M-CSF-dependent signaling” and “Cellular mechanisms” when the proteomes of osteoclasts derived from bone marrow macrophages (BMMs) and RAW264.7 cells where compared.

### Metabolomics

Metabolomics is the study of end products derived from complex biochemical reactions including small molecules such as sugars, lipids, amino acids, organic acids, nucleotides, and steroids. Like proteins, metabolites cannot be amplified, and thus must be detected at endogenous levels. The chemical diversity and large range of abundance (< 2000 metabolites) creates unique challenges when detecting and quantifying individual metabolites, and thus demands more sophisticated equipment. Two technologies are mainstay for metabolomics: (i) mass spectrometry and (ii) nuclear magnetic resonance (NMR) spectroscopy. For mass spectrometry–based metabolomics, samples are typically subject to harsh solvent extraction methods prior to liquid-chromatography separation and tandem mass spectrometry [32]. In comparison, NMR-based metabolomics represent a non-destructive and rapid detection method that enables the re-use of samples, which is advantageous when performing large-scale screening or when in vivo studies ensue.

While the application of metabolomics has recently gained momentum for biomarker discovery of osteoporosis [33–39], its implementation in fundamental bone biology remains comparatively scarce. Nonetheless, there are a few recent examples in the literature employing metabolomics to monitor the metabolic footprint during stromal cell-to-osteoblast differentiation [40], analyze the extracellular metabolic changes in mechanically stimulated osteocytes [41] and, to compare the metabolomics signatures of extracellular vesicles derived from osteoclasts and odontoclasts [42].

### Trans-omics in Bone Biology and Disease

Compared with single omics and multi-omics analyses that integrate two omics data sets, trans-omics approaches that integrate data sources from at least three individual omics layers provide richer information about the state of a tissue or cell [3, 43, 44]. When coupled with bioinformatics and computational workflows, trans-omics not only reveals information of a single regulatory layer, but also the intertwined relationships between layers (Fig. 1). Being “the sum of their parts,” integration of multiple molecular layers therefore enables bone biologists to generate multi-dimensional molecular blueprints of bone, and its resident cells, to identify and trace molecular pathways and reveal mechanistic interactions. Although the importance of trans-omics has gained rapid appreciation in recent years, successful integration of more than two omics datasets remains rare in bone research. Therefore, we herein highlight integrative multi-omics studies that apply the principles of trans-omics to unmask new molecular pathways and interactions that have recently advanced our mechanistic understanding of bone biology and skeletal disease.

### Multi-omics to Study Osteoblast Differentiation, Osteoblast-to-Osteocyte Transition and Osteogenesis

Recent studies have applied integrated analysis of multi-omics data to unveil molecular cascades regulating the differentiation of bone marrow mesenchymal stem cells (BMMSCs) into adipocytes, osteoblasts, and osteocytes. For example, Salmi et al. [45] performed multi-omics bioinformatics analyses to decipher genes and networks regulating adipocyte-induced trans-differentiation of osteoblasts. By integrating the adipocyte secretome together with the osteoblast transcriptome, the authors unveiled a total of 271 physical molecular interactions and unmasked the PI3K-AKT, the JAK2-STAT3, and the SMAD pathways as key signaling hubs driving adipocyte-induced trans-differentiation of osteoblasts. Similarly, Pihlström et al. [46] combined RNA-seq, proteomics, and phosphoproteomics to characterize the trans-differentiation of human dermal fibroblasts into osteoblast-like cells. Surprisingly, cross-correlation of phosphoproteomics data revealed only a single phosphorylated protein (i.e., hyper-phosphorylation of promyelocytic leukemia protein, PML) differentially expressed between BMMSCs and fibroblasts. Recently, Teng et al. [47] combined RNA-seq, assay for transposase-accessible chromatin sequencing, and high-throughput chromosome conformation capture (Hi-C) to monitor spatial chromatin conformation dynamics and the relationship between chromatin accessibility and gene expression during BMMSC differentiation into adipocytes and osteoblasts. By integrating these multi-omics data, together with data sets from GWAS, eQTL, and motif analyses, the authors identified 274 genes and 3634 SNPs associated with bone degeneration and osteoporosis.

Transition of osteoblasts-to-osteocytes results in progressive changes in cell morphology from a cuboidal to a stellate shape, a process that requires coordinated regulation and interplay between transcriptional and epigenetic mechanisms, such as histone modifications (e.g., H3K27 trimethylation). Using an integrative genome-wide transcriptomic (RNA-seq) and epigenomic (ChIP-seq) approach, Xia et al. [48•] recently profiled the expression of genes and epigenetic modifications during osteoblast-to-osteocyte differentiation of IDG-SW3 cells. Transcriptomics analyses identified 1239 osteocyte signature genes, 318 (including Sost, DMp1, Mepe, and Dkk1) of which were upregulated during osteocyte differentiation. Parallel genome-wide analysis of H3K27me3 by CHIP-seq revealed a concomitant decrease in H3K27me3 signals in the same gene cluster implying that H3K27me3 regulates the expression of genes required for osteocyte differentiation. By integrating these data together with robust computational and informatics analyses, the authors discovered that Utx, a H3K27 demethylase, binds to regulatory regions of critical osteocyte genes. The physiological importance of Utx was validated in mice by conditionally deleting Utx in osteocytes, which corresponded with a low bone mass phenotype associated with decreased osteocyte numbers. Based on these findings, the authors concluded that Utx positively regulates osteoblast-to-osteocyte differentiation through H3K27me3 modifications in osteocyte genes. Overall, this study serves as an exemplar of multi-omic integration to identify and connect new molecular mechanisms that regulate osteoblast-osteocyte transition.

As mechanosensing cells, osteocytes sense and respond to mechanical stimuli that are applied to bone. Thus far, only one study by Santos et al. [49] has employed integrative multi-omics to examine the effects of mechanical load on osteocyte signaling. By combining a computer-controlled bioreactor that mimics exercise with RNA-seq and tandem liquid chromatography–mass spectrometry (LC–MS), the authors mapped the transcriptome and secretome of mechanically stretched human osteocytic cells. Remarkably, the authors found that a single cyclic stretch was sufficient to elicit activation of a network of signaling pathways including extracellular matrix remodeling, cell–matrix interaction, bone remodeling, and cancer. Based on these analyses, the authors concluded that mechanically stimulated osteocytes support bone regeneration via ossification extracellular matrix remodeling and may simultaneously modulate cancer through transcriptional activity linked to genome integrity and extracellular matrix remodeling.

Lastly, in an effort to advance our understanding of de novo matrix formation and mineralization, Bergen et al. [50] recently integrated RNA sequencing with genomic data sets to define the transcriptomes of ontogenetic and regenerating zebrafish scales. Although zebrafish are often dismissed as being too evolutionary distant from humans, by combining orthologous gene mapping and hypergeometric tests the authors showed that differentially expressed genes (604) were strongly enriched for human orthologues related to human musculoskeletal diseases including monogenic skeletal disorders (e.g., osteogenesis imperfecta) and polygenetic skeletal traits (including eBMD and height). Importantly, two of these genes, i.e., COL11A2 and SPP1, were functionally validated using zebrafish mutants and displayed exoskeletal and endoskeletal features consistent with genetic association predictions. Overall, this study highlights how integrating interspecies omic data sets derived from lower vertebrates with omics data derived from humans’ holds potential for the discovery of evolutionary conserved osteoanabolic mechanisms.

### Integrated Multi-omics to Unveil Mechanisms Regulating Osteoclast Formation, Fusion, and Function

Being multinucleated giant cells firmly adhered to bone, primary osteoclasts are notoriously difficult to isolate intact from bone and their sheer cell size precludes their isolation by single-cell methods, at least for *bona fide* mature osteoclasts (i.e., 5-day RANKL cultures) whose diameters average upwards of 250 μm, beyond the dimensions of conventional cell sorting nozzles (i.e., ~ 70 to 100 μm). As such, until very recently, most osteoclast -omics studies have relied on bulk profiling methods using osteoclasts differentiated on plastic in vitro. Early examples of integrative analyses using osteoclast omic datasets were relatively simple and compared protein expression profiles obtained using proteomic methodologies (e.g., 2D DIGE) with mRNA expression analyzed by DNA microarrays from “osteoclast-like cells” derived from RANKL-stimulated RAW264.7 macrophages [51, 52]. Advancements in omics technologies have recently spurred re-evaluation of correlative gene/protein expression analysis during RANKL-mediated osteoclastogenesis, including at the single-cell resolution [53], unveiling new regulatory hubs important for osteoclast lineage commitment and the establishment of a multinucleated cell state.

For example, Caputo et al. [54] recently combined RNA-seq with ChIP-seq to investigate initiating regulatory events leading to osteoclast lineage commitment in response to RANKL. Integration of these datasets together with with assay for transposable accessible chromatin by high-throughput sequencing (ATAC-seq) data enabled the authors to construct transcriptional factor-focused gene regulatory networks which revealed both positive and negative feedback loops of transcription factor cross-regulation and identified Myc as the central transcription factor regulating this process. Further analyses confirmed that Myc positively regulated key pro-osteoclastic transcription factors (NFactc1, Fosl2m, and Nfkb2) while simultaneously repressing the macrophage transcriptional program (Irf8, Mafb, Spi1/Pu.1). Overall, the integration of these omic studies uncovered cooperative role(s) for Brd2/4 with Myc to orchestrate early chromatin events and transcription factor regulatory networks that determine osteoclast lineage commitment. Studies by Guérit et al. [55] combined quantitative proteomics (SILAC) with RNA sequencing to compare the global proteomes and transcriptomes of primary mouse osteoclasts and dendritic cells. Using correlation, GO term enrichment and protein–protein interaction analysis, the authors uncovered a number of protein/signaling networks that distinguish the lineage specification of each myeloid derived cell-type. In addition, the authors identified 38 proteins of unknown function in osteoclasts, half of which were functionally validated using a siRNA-based screen, including Tubb6, a β-tubulin isotype uniquely expressed in mature osteoclasts that was functionally required for podosome patterning.

By taking advantage of an established *trans*-regulated gene network (i.e. MMnet, macrophage multinucleation network) derived from transcriptional profiling of primary macrophages isolated from inbred rats that feature spontaneous fusion [56] and mapping eQTLs in fusing macrophages, Pereira et al. [57•] recently identified a *trans*-regulated gene co-expression network (consisting of 190 genes) that were enriched for osteoclast genes as well as GWAS variants associated with bone-related phenotypes. By combining this regulatory network with a rapid-throughput skeletal phenotyping pipeline and gene knock-down studies in human osteoclasts, the authors demonstrated that the central network gene *Bcat1* (as well as 7 out of 13 co-regulated MMnet genes) were *bona fide* regulators of osteoclast multinucleation and bone mass. Although not explicitly an example of trans-omics, this study serves as an excellent example of how pairing existing transcriptomic data from rodents with systems genetics in humans can help connect cell-type specific regulatory networks with genotype–phenotype associations’ years after their collection. In addition, it further illustrates the importance of supplementing omics studies with functional validation in model organisms and physiologically relevant cells, and thus stands as an exemplar of multi-data integration to connect mechanisms in keeping with the principles of trans-omics.

In addition to exploring drivers of RANKL-induced osteoclast differentiation and fusion, there are also several recent examples using integrative multi-omic analyses to uncover mechanisms related to changes in osteoclast resorptive activity in mice with pronounced bone phenotypes. For example, Arandjelovic et al. [58] combined transcriptomics with proteomic approaches to investigate how loss of the cytoplasmic adapter protein ELMO1 protects against osteoclast-mediated bone destruction in mouse models of osteoporosis. By analyzing differentially expressed genes in osteoclasts derived from wild-type and Elmo1-deficient mice, the authors uncovered changes in the expression levels of several genes linked to human diseases including small GTPases and cell motility regulators, as well as genes involved in extracellular enzymatic degradation with potential relevance to osteoclast function in bone demineralization and inflammation. By coupling this data with mass spectrometry and STRING analysis, the authors further showed that Elmo interacted with several cellular receptors and subunits of the V-ATPase acidification complex. Based on these combined omics data sets, together with detailed observations in preclinical models of osteoporosis and arthritis, the authors concluded that ELMO1 functions as part of a signaling network that regulates osteoclast function and bone loss.

Finally, a recent paper by Ng-Ribet-Guo et al. [59] employed organellomics to map the molecular landscape of osteoclast secretory lysosomes. The authors identified 218 secretory lysosome residents, including the membrane transport protein Slc37a2, whose human orthologue gene was found to harbor a genome-wide significant signal for eBMD [13••]. Accordingly, deletion of Slc37a2 in mice resulted with a profound high bone mass phenotype that featured an accumulation of dysfunctional osteoclasts. To define the mechanistic basis for this osteoclast deficit, the authors performed multi-omics analyses (transcriptomics, proteomics, and metabolomics) on osteoclasts derived from primary BMM from Slc37a2-deficient and wild-type mice. Whereas comparative analyses of proteomic and transcriptomic data revealed very few overlapping proteins/genes including Slc37a2, metabolomics revealed that monosaccharide sugars (i.e., glucose and fructose) were overrepresented in Slc37a2-deficient osteoclasts, implying that Slc37a2 functionally regulated the transport of monosaccharides. This position was confirmed using live cell imaging to monitor the export of monosaccharides from osteoclast secretory lysosomes. Based on these findings, the authors concluded that Slc37a2 is a sugar transporter on secretory lysosomes that is required for osteoclast function, and thus the maintenance of homeostatic bone mass. This study demonstrates how integration of multiple molecular omic layers can be used to decipher disease mechanisms and lends further support to the value of pairing human and mouse datasets to discover new and physiological-relevant regulators of skeletal bone mass.

### Trans-omics in Bone Diseases

Accumulating studies in recent years have also indicated that the integrated analysis of multi-omics data may prove useful towards our understanding of the pathobiology of bone and joint diseases such as osteoporosis [60••, 61] and rheumatoid arthritis (RA) [62], and the identification of biomarkers to aid patient diagnosis. For example, Qiu et al. [63••] identified novel osteoporosis biomarkers from genomic, transcriptomic, DNA methylomic, and metabolomic data curated from 61 and 58 individuals with high and low BMD respectively. By integrating and connecting these data sets, the authors established a multi-omics biomarker panel, consisting of differentially expressed genes, methylation sites, and metabolites, for discriminating high and low BMD groups. They also identified a large number of QTLs for these biomarkers, some of which have previously been identified as associated with eBMD [13••]. Similarly, Ding et al. [64] identified diagnostic biomarkers for two distinct groups of RA patients by performing metabolomic and transcriptomic profiling of serum, urine, synovial fluid, and synovial tissue samples obtained from RA patients and integrating these data with clinical phenomes and experimental rodent models mimicking arthritis.

There are also recent examples of the implementation of integrated multi-omics to elucidate the pathogenesis of bone loss associated with chronic kidney disease (CKD) (i.e., renal osteodystrophy, ROD). By building upon the clinical observation that the transcription factor HNF4a2 expression is suppressed in patients with CKD, David and co-workers [65] performed multi-omics analyses of bones and cells lacking or overexpressing Hnf4α1 and Hnf4α2. These omic studies revealed that HNF4α2 is the main osseous Hnf4α isoform that regulates osteogenesis, and can protect against bone loss in mice with CKD when Hnf4α2 is specifically overexpressed in osteoblasts. Based on these data, the authors concluded that osseous HNF4α2 deficiency contributes to the pathogenesis of ROD, and thus may explain intrinsic bone defects observed in patients with CKD.

In comparison, to systematically explore the molecular landscape of autosomal dominant osteopetrosis type II (ADO2), Li et al. [66] combined multi-omics with bioinformatics analysis to establish a multi-omics landscape of reprogrammed ADO2-iPSCS harboring a mutation in CLCN7 (R286W). Using whole-genome re-sequencing, DNA methylation, and N6-methyladenosine (m6A) analysis together with previously published transcriptome and proteome datasets, they observed epigenetic changes in DNA methylation levels that correlated inversely with expression levels of genes involved in osteoclast differentiation and the p53 signaling pathway suggesting that the pathogenesis of ADO2 may begin much earlier in life than originally anticipated. Finally, De Ridder et al. [67] used a multi-omics approach (genomics (WGS) and RNA-seq) to unravel the genetic cause of four unrelated patients with melorheostosis, a rare sclerosing bone dysplasia characterized by asymmetrical and progressive cortical hyperostosis. Although the final integrative analysis was confined to a single patient owing to limited sample availability, this study demonstrates the value added using multi-omics to investigate the genetic basis of rare bone diseases.

In summary, the abovementioned studies illustrate the power and utility of integrating multiple omic layers at different regulatory levels to connect and unveil new interlinked pathways and networks that can be used to advance our fundamental understanding of bone cell biology as well as uncover new disease mechanisms and biomarkers to aid patient diagnosis. In addition, they highlight the value of combining interspecies omics data sets together with functional studies in cells and model organisms to validate new regulators of bone mass, which, in turn, may serve as potential targets that can be exploited therapeutically for the treatment of bone diseases.

## Challenges and Future Perspectives

While the era of integrative multi-omics/trans-omics is poised to revolutionize the bone field, it also brings new challenges [68]. First, storing and handling “Big Omic” data sets, which can generate tera- to penta-byte-sized files daily, is a considerable challenge that will demand substantial computational grunt and support. Second, unifying and interpreting multi-level datasets requires sophisticated statistical and analytical tools (e.g., R-based software packages such as integrOmics [69], mixOmics [70], correlation-based local approximation of membership [71]), many of which lie beyond the informatics expertise of an individual research group. Therefore, coordinated interdisciplinary efforts between bone biologists, computational biologists, biostatisticians, and biomathematicians will be needed in order to integrate multi-dimensional data into a biologically meaningful context. In this regard, leveraging artificial intelligence and deep learning systems, which can dynamically generate, collect, integrate, and analyze large-scale multi-omics data in a timely manner will become of increasing importance in the future. Similarly, as we continue towards open and transparent research practices, it will equally be important to incorporate and consolidate large multi-omic datasets from humans, cellular experiments, and model organisms into central public repositories such as the Musculoskeletal Knowledge Portal (http://mskkp.org/) [72] to enhance data accessibility and its utility by the bone community.

There are also a number of biotechnological limitations inherent in existing omic technologies that need to be considered and overcome. For example, while traditional bulk omics provide an overview of the average information of a cell population or targeted tissue it does not lend information regarding individual cell types or activation states. Single-cell sequencing data sets that profile bone cell heterogeneity, while defining intermediate cell states (e.g., [53]), will therefore need to be integrated. Furthermore, neither bulk omics nor single-cell sequencing data provides spatial information, which is required to fully understand the complexity and heterogeneity that exists within the bone microenvironment [73, 74]. Therefore, additional layers of spatial and temporal information will need to be gathered from spatial technology platforms [75] such as sequencing-based techniques (e.g., spatial RNA sequencing) or imaging based–technologies including multiplexed fluorescence in situ hydridization (FISH), mass spectrometry imaging (MSI), imaging mass cytometry (IMC), as well as emerging technologies such as correlative nanoscale secondary ion mass spectrometry (NanoSIMS) [76] to enable researchers to match omics data precisely to its bone resident cell(s) in situ.

While trans-omics can reveal a multitude of mechanistic regulatory relationships between molecules, precisely how each network regulates bone cell differentiation, function, and/or contributes to bone homeostasis also requires functional validation. In this regard, an important future avenue will be to supplement multi-omic studies with functional and phenotypic screens (e.g., functional genomic screens using CRISPR/siRNA-based libraries) either in bone cells cultured in vitro or using vertebrate models (e.g., mice and zebrafish) [2]. Finally, how to condense complex multi-layered trans-omics data into a simple yet meaningful clinical tool to aid diagnosis of patients with bone diseases, and thus guide more personalized treatment options remains a significant future challenge.
